# Too precarious to walk: an integrated “three delays” framework for modeling barriers to maternal health care and birth registration among stateless persons and irregular migrants in Malaysia

**DOI:** 10.1186/s41118-021-00129-3

**Published:** 2021-09-03

**Authors:** Amanda R. Cheong, Mary Anne K. Baltazar

**Affiliations:** 1 Department of Sociology, The University of British Columbia, 6303 NW Marine Drive, Vancouver, BC V6T 1Z1 Canada; 2 Faculty of Humanities, Art, and Heritage, Universiti Malaysia Sabah, Kota Kinabalu, Malaysia

**Keywords:** Legal identity, Infant and maternal health, Reproductive health, Birth registration, CRVS systems, Statelessness, Irregular migration, Sabah, Malaysia

## Abstract

This study extends Thaddeus and Maine’s (1994) “three delays” framework to model the interrelated barriers to maternal health care and birth registration. We focus on stateless persons and irregular migrants, populations that are especially at risk of being “left behind” in United Nations member states’ efforts to “provide legal identity to all” as part of the 2030 Sustainable Development Agenda. Drawing on qualitative fieldwork conducted in Sabah, Malaysia, we model delays in accessing maternal health care and birth registration as an integrated, cyclical process. We identify the political and legal barriers that stateless or migrant families confront while deciding to make institutional contact (Phase I), identifying and reaching health or registering institutions (Phase II), and receiving adequate and appropriate treatment (Phase III). We find that exclusion from one system raises the risk of exclusion from the other, resulting in a range of negative consequences, including increased health risks, governments’ impaired ability to monitor population health, and the perpetuation of intergenerational cycles of legal exclusion.

## Introduction

In 2016, the United Nations made it a target “to provide legal identity to all, including birth registration” by 2030 as part of its Sustainable Development Goals (SDG) agenda, which succeeds the Millennium Development Goals of 1990–2015. In many respects, the achievement of universal birth registration is key to actualizing the SDGs’ pledge to “leave no one behind.” For one, the SDGs explicitly prioritize birth registration, because it provides individuals with their first—and often their most fundamental—form of legal identity, defined herein as the recognition of one’s personhood before the law (Dahan & Gelb, 2015). Governments also rely on data from civil registration and vital statistics (CRVS) systems to inform their planning and other resource allocation decisions, as well as to measure their progress along a number of development indicators (AbouZahr et al., 2015; Jackson et al., 2018). Furthermore, in many contexts, expanding access to birth registration means extending official recognition to—and thus encouraging the political, social, and economic integration of—historically marginalized or disenfranchised populations (Setel et al., 2007). Yet, despite the importance of birth registration for establishing the right to a legal identity, along with other fundamental rights, it is estimated that the births of 1 in 4 children under the age of five worldwide have not been recorded (UNICEF, 2020a).

This article contributes to a growing body of research investigating the proximate determinants and consequences of the under-registration of vital events. We focus on an important factor that has been recognized as both a facilitator and outcome of birth registration: mothers’ healthcare institutional contact during pregnancy and birth. Encouraging the use of formal healthcare providers is a widely recommended intervention for improving both maternal and infant health as well as governments’ ability to monitor them (Onagoruwa & Wodon, 2021; Siagian et al., 2019; World Health Organization, 2014). Healthcare personnel are often the first official witnesses to a child’s birth, and in many contexts are relied on as informants to government bodies about the circumstances of the vital events of births and deaths (Fagernäs & Odame, 2013). Initiatives have been underway in many countries to more closely integrate healthcare and civil registration systems (Jackson et al., 2018; Muzzi, 2010). In turn, birth certificates function as the documentary bases for obtaining other types of official identification which are routinely required by clinics, hospitals, and other healthcare providers. The achievement of SDG target 16.9 (“provide legal identity for all”), therefore, is interrelated with a number of other targets, including 3.1 (“reduce the global maternal mortality ratio to less than 70 per 100,000 live births”) and 3.2 (“end preventable deaths of newborns and children under 5 years of age”).

We examine the interconnections between maternal health care and birth registration access by modeling how they may operate within a demographic that is at particularly high risk of being “left behind” in UN member states’ efforts to meet the SDGs: stateless persons, irregular migrants, and their descendants (whom we refer to on the whole as “legally marginalized persons,” henceforth abbreviated as LMPs), using Sabah, Malaysia, as a case study. A stateless person is defined by the 1954 Convention Relating to the Status of Stateless Persons as someone “who is not considered as a national by any State under the operation of its law.” We use the term “irregular migrants” to refer to people who engage in movement across an international border outside of the legal frameworks of their sending, transit, or receiving countries (International Organization for Migration, 2019). Specific scholarly attention to LMPs is warranted given that they may face barriers obtaining, maintaining, or substantiating their legal identities due to the denial, invalidation, expiration, or dispossession of official documentation or registration. Furthermore, a large literature has drawn attention to disparities in access to health care between citizens and non-citizens—particularly those who are undocumented—across a wide range of national contexts (Cuadra, 2012; Gelatt, 2016; Grit, et al., 2012; Hacker et al., 2015; Korinek & Smith, 2011; Ortega et al., 2007; Siddiqi et al., 2009). More specifically, a lack of legal status has also been found to be associated with adverse health outcomes among migrants (Asad & Clair, 2018; Cavazos-Rehg et al., 2007; Cheong & Massey, 2019), including their birth outcomes  (Reed et al., 2005).

To conceptualize the interrelated barriers to maternal health care and birth registration among LMPs, we draw upon and extend the “three delays” framework created by Thaddeus and Maine (1994). This framework was originally designed to theorize the factors influencing maternal mortality outcomes following the onset of an obstetric complication. Since its conception, it has proven to have wide applicability across demographic groups, regional contexts, and substantive concerns. The framework has since been applied beyond emergency obstetric care-seeking to preventative healthcare behaviors, such as the use of health facilities or skilled attendants for prenatal care and delivery (Gabrysch & Campbell, 2009). It has also been used to analyze maternal health-seeking behaviors within specific demographics, including immigrant women (Binder et al., 2012; Esscher et al., 2014), forming part of a broader body of work examining maternal healthcare access among immigrants in various national contexts (Almeida et al., 2013; De Freitas et al., 2020). Most directly relevant to this study, Bennouna et al. (2016) adapted the three delays framework to understanding barriers to accessing civil registration in Indonesia. We take their effort to export the framework beyond the context of maternal healthcare access as the departure point for this study. We adapt the framework to model barriers to maternal health care and birth registration as integrated cyclical phenomena. Focusing on the experiences of LMPs in Sabah, Malaysia, we highlight the roles of state policies and legal status in shaping the maternal mortality and civil registration coverage outcomes of the populations that reside within their borders.

Our extension of the three delays framework thus offers novel theoretical utility in two main respects. First, we recognize that access to government services is routinely stratified on the basis of legal status. We focus on the case of LMPs in the Malaysian context, because this population exemplifies the need to consider legal and political causes of delays to accessing both maternal health care and birth registration. We demonstrate how state policies both directly and indirectly stratify access to rights and benefits—namely, a legal identity and health care. In doing so, we expand the consideration of determinants of access to health and registering institutions beyond the traditional dimensions of socioeconomic and cultural factors.

Second, we model delays in accessing maternal health care and birth registration as an integrated cyclical process rather than as two discrete spheres, in effect combining the respective foci of Thaddeus and Maine (1994) and Bennouna et al. (2016) into a single framework. As we demonstrate in our case study of Malaysia, contact with official healthcare providers at the time of pregnancy and labor is crucial for obtaining documentary proof for substantiating the circumstances of a child’s birth. Correspondingly, birth registration can function as, and provides the documentary basis for obtaining, valid proof of identity, which is typically required for admission into formal healthcare institutions. Proof of parentage or birth within a territory may also be factored into the practical determination of one’s citizenship, the holding of which may unlock eligibility to subsidized or even free health care in some national contexts. Therefore, exclusion from one system increases the likelihood of exclusion from the other, with the potential to create an intergenerational feedback loop of deprivation of legal identity and its associated rights.

This paper is organized as follows. First, we describe our study context, data, and methods, including an elaboration of our document inventory interview method. We then introduce in detail our integrated three delays framework, describing how it builds upon and extends the work of Thaddeus and Maine (1994) and Bennouna et al. (2016). Then, in discussing our findings, we focus especially on how state policies and legal status shape delays at each stage of the healthcare-seeking and registration process. We conclude with policy recommendations for improving access to legal identity and basic rights, including maternal health care, among LMPs in Sabah and provide guidance about generalizing our integrated three delays model beyond the Malaysian context.

### Study context

Data for this study comes from fieldwork conducted between 2016 and 2017 in Malaysia, an upper middle-income country in Southeast Asia with an official population of 32.7 million, according to 2020 estimates (Department of Statistics Malaysia, 2020). Having undergone rapid economic development since the 1970s, Malaysia has made significant advances in improving its population health. For example, Malaysia’s reported infant mortality rate in 2019 was 7.2 infant deaths per 1000 live births, down from 67.2 in 1960 (Department of Statistics Malaysia, 2020). This 2019 estimate is almost half that of the average for upper middle-income countries (11.6) and nearly five times as low as the global average (29.4) (UN Inter-agency Group for Child Mortality Estimation, 2019). The government’s latest reported maternal mortality ratio (MMR) is 22.7 in 2014 (preliminary estimate). According to UN estimates, this figure is 40 (as of 2015)⁠. This compares to the global MMR of 216 and an upper middle-income MMR of 41. East Asia and Pacific is 59 as of 2015 (63, excluding high-income countries).

Impeding further progress is the reality that non-citizens and specific racial minorities are still being “left behind” in Malaysia’s journey toward achieving its SDG targets. Malaysia makes for an ideal case study for studying the interconnections between registration status and maternal health because access to rights is heavily stratified by legal status. In particular, entitlement to government-subsidized health care is largely denied to the significant population of non-citizens living within its territory. Nationally, 3 million people residing in Malaysia are officially identified as non-citizens (Department of Statistics Malaysia, 2020). The easternmost state of Sabah, where the majority of this research was conducted, has the highest ratio of citizens to non-citizens: nearly 1–3 (Department of Statistics Malaysia, 2020), with the non-citizen population composed predominantly of persons of Indonesian and Filipino descent. These figures, however, may not take into account the significant yet unknown number of LMPs—stateless persons, irregular migrants, and their descendants—residing in the country who not only are left unaccounted for in official statistics but also are potentially deprived of a legal identity. Statelessness is a “homegrown” problem in Malaysia in that it is not only experienced by migrants and refugees, but also by several categories of people born and raised in Malaysia (Liew, 2019). One major risk factor for statelessness in Malaysia is the lack of vital documentation, particularly birth certificates. Though birth registration is touted by the Malaysian government as being complete at the national level, the quality and completeness of birth registration is uneven, particularly within the country’s poorest state of Sabah (Lai and Tey, 2021).

Under-registration in Sabah is more than a problem of lack of resources or technical capacity; it is intimately tied to questions of national identity and belonging (Cheong, 2019). Located in northern Borneo, the state of Sabah is characterized by migratory ties within the region that predate the imposition of colonial and postcolonial political boundaries (Koh, 2017). Sabah’s economy is highly dependent on migrant labor from Indonesia and the Philippines, which takes both authorized and unauthorized forms (Dollah & Abdullah, 2018). Sectors that commonly rely upon migrants include agriculture (particularly the palm oil industry), construction, and domestic service (Dollah & Abdullah, 2018; Pye et al., 2012). Despite the state’s reliance on migrant labor, anti-immigrant sentiment is high in Sabah and has come to be expressed through the politicization of official documents, including birth certificates and national identity cards (Sadiq, 2010). Contestations over documentation date back to the 1990s, when it was alleged that the ruling United Malays National Organization had fraudulently issued identity cards to immigrants in exchange for electoral support (known as *Projek/*Project *IC*).^1^ Irregular migrants are commonly vilified and scapegoated by politicians and the mass media (Kassim, 2009), in which they have become associated with the theft of domestic jobs, criminality, and—particularly with the COVID-19 pandemic—disease (Baltazar & Cheong, 2021; Wahab, 2020). It is within this context of tense racial and economic conflict and popular anxieties over the demographic identity of Sabah that LMPs struggle to obtain vital documents, a legal identity, and even citizenship (Cheong, 2019).

Existing research on the intersections between health and registration status in Malaysia, and in particular Sabah, is scant. In their review article, Lasimbang et al. (2016) lamented the lack of available data on migrants’ sexual and reproductive health in Sabah. A scoping review conducted by de Smalen et al. (2021) of studies published between 1965 and 2019 on the health of migrants in Malaysia similarly noted the dearth of availability of high-quality evidence on the topic. That being said, there is a broad recognition of the multiple exclusions and vulnerabilities faced by LMPs in Malaysia, including in the realms of health and access to health care. Legal scholars have identified the multiple legal and administrative pathways that lead to statelessness in both West and East Malaysia, as well as the health care and other rights deprivations that are associated with the denial of citizenship (Liew, 2019; Mohamed Razali, et al., 2015). Anthropological and ethnographic work has explored the lived experiences of stateless communities in Sabah—such as the Bajau Laut and children of Indonesian and Filipino descent—showing how their invisibilization in the eyes of the Malaysian state facilitates their exclusion from rights, resources, and recognition (Acciaioli, et al. 2017; Allerton, 2020; Lumayag, 2016). Our research builds upon these literatures by providing ethnographically informed insight into the health and healthcare-seeking behaviors of LMPs in Sabah. In doing so, we show how civil registration status operates as both a determinant and an outcome of healthcare institutional contact, owing to its instrumentality in establishing an individual’s legal identity, including citizenship status.

#### Legal frameworks and administrative procedures for birth registration in Sabah

Civil registration in Peninsular Malaysia is governed by the *Births and Deaths Registration Act 1957 (Revised 1983)*, which mandates that “the birth of every child born in Malaysia shall be registered.”^2^ Sabah, as with its fellow Eastern Malaysian state of Sarawak, is governed by its own *Registration of Births and Deaths Ordinance of 1951*, which also makes no distinction as to the rights and obligations to register the births of citizens versus non-citizens. Civil registration and identity documentation matters in Malaysia are overseen by the National Registration Department (Jabatan Pendaftaran Negara in Bahasa Malaysia), which was established in 1948 under the Ministry of Home Affairs and which implements the administrative procedures for registering births in Peninsular Malaysia, Sabah, and Sarawak.

In Malaysia, the onus for registering a birth is solely demand-driven, meaning that it is up to the parent, legal guardian, or “person having knowledge of the birth” to approach a National Registration Department office when a child is born. A successful registration of birth is dependent not only on whether an applicant reports its occurrence at a National Registration Department office, but also on the provision of necessary supporting documentation. In Sabah, a “normal registration of birth,” which occurs within 14 days of birth,^3^ requires the following supporting documentation, summarized in Table 1 (Jabatan Pendaftaran Negara, 2020):

**Table 1:** Documents required by the National Registration Department for normal registration of birth in Sabah

Child	Parents
Form A and N1 (extract) completed	
Confirmation of birth form from hospital or certification of home birth from midwife/doctor	Identity cards or entry permits or passports of the parents and the person reporting the birth
Prenatal card (maternal examination book)	Marriage or divorce or death certificates of the parents
Child’s health and immunization card	

Source: Jabatan Pendaftaran Negara, 2020

The two forms issued by the National Registration Department at the time of registration are Form A and Form N1. Form A is the Citizenship Application Form (Borang Permohonan Kewarganegaraan in Malay), which is issued in accordance with the Federal Constitution Articles 15(1), 16, and 16A.^4^ Form N, the Application for the Extraction of a Birth Certificate (Permohonan Carian/Cabutan Daftar Kelahiran in Bahasa Malaysia), is issued in accordance with the *Sabah Registration of Births and Deaths Ordinance of 1951*.^5^

Applicants are also required to produce forms that are issued by formal healthcare providers. These include the confirmation of birth form from the hospital, issued by the doctor, or certification of home birth from the midwife who attended the birth. The confirmation of birth contains information about the date and time of birth, the child’s weight and height, sex, method of delivery, and details about the doctor or midwife. Additionally required is a prenatal card : also known as a maternal examination book, which is issued by both public and private health facilities in Malaysia, this card tracks information about both mother and baby throughout the pregnancy stage. Finally, the applicant must also furnish the child’s health and immunization card, which contains information about the date of immunizations received, provided by the health worker who administered the immunizations.

Documents are required from both biological parents. If the parents are Malaysian citizens, they must produce their MyKads, the national identity card that is compulsory for all Malaysian adult citizens to hold. Non-citizen parents are otherwise asked to provide their valid passports and entry permits. They are also required to provide a marriage certificate, divorce certificate, or death certificate(s) to determine the legitimacy of the child.

The successful registration of a birth is, therefore, dependent on the acquisition of a number of documentary prerequisites. The complete set of medical documents can be obtained only through regular and sustained contact with a recognized healthcare provider throughout the prenatal and postnatal period, and not only during the event of birth itself. Furthermore, despite the legal mandate to register every child born within Malaysia, non-citizens are required to furnish their passports and entry permits, which effectively renders parents’ authorized status a prerequisite for the child’s recognition of birth. The National Registration Department’s complex and strict administrative requirements for registering a child’s birth create several opportunities for non-registration to occur, with non-citizens and LMPs being at a pronounced disadvantage.

## Data and methods

This study is based on ethnographic observations and 105 household interviews conducted with LMPs who faced challenges applying for vital documents and citizenship. This included undocumented migrants, asylum seekers, as well as undocumented and/or stateless persons who were born in Malaysia but were denied citizenship and/or the documentation to prove their claims. Interviews were conducted using an interviewing instrument that we call a “document inventory.” A document inventory is a qualitative, semi-structured interview framework that prompts families to take stock of the material evidence they possess to substantiate their legal identities and existence. We explored how families’ lives were shaped by documents over the course of a semi-structured interview about the household’s migration histories, everyday experiences living in Malaysia, the limitations and challenges they face, and the nature and frequency of their interaction with government institutions (see “Appendix” for the sample interview guide topics). The document inventory method bears many similarities to the process of capturing a household’s family history, albeit with documentation serving as the central focus of conversation. For households with children, special attention was paid to the use of maternal health services before, during, and after pregnancy. Document inventories bear a closer likeness to oral histories than to closed-ended surveys in that they are flexible and open ended rather than prescriptive. By using them, the objective is not to derive a statistical portrait of a population’s documentation status based on a number of standardized variables, but rather to produce in-depth narratives about the practices, processes, and beliefs that people engage in and hold with regard to official documents and the impacts that documents have on their lives.

There are a number of methodological advantages to conducting document inventories in the form of semi-structured narrative interviews compared to closed-ended surveys: by cataloguing a household’s family history through the lens of documentation, researchers are able to empirically convey registration as a complex social process, and to identify and map the multiple stakeholders involved. The semi-structured, open-ended format of the document inventory helps researchers to avoid imposing preconceived assumptions about individuals’ attitudes and relationships toward documentation, and to accommodate diverse and complex life course and family formation trajectories. The document inventory approach also allows us to explore multiple dimensions of individuals’ relationships to registration and documentation, including the extent to which a particular household is incorporated into government registration systems, the challenges members faced accessing registration or documentation, the meanings and values they ascribe to documents, and the impacts of being unregistered or undocumented on their lives.

For LMPs in Sabah, our document inventory modules cover the following topics: demographic and background information about household members (including age, race/ethnicity, religion, languages spoken, marital status, occupational histories, migration histories); documentation (including processes for obtaining and attitudes toward documents possessed, reasons for not having desired forms of documentation, how documentation or lack of documentation impacts everyday life); and experiences and attitudes with regard to citizenship, migration, legal status, and residence.^6^

Before proceeding with a discussion of our findings, we provide a brief overview of the demographic characteristics of the sample under study. Here, we report the characteristics of mothers and their children, given this study’s focus on maternal health. Of the 105 households captured during our fieldwork, 87 contained women who had at least 1 child, and 5 contained women who were pregnant at the time of the first interview.^7^ While the majority of interviews were cross-sectional, we were able to conduct one or more follow-up interviews, or additionally collect ethnographic observations of families as they navigated various registration or application processes, in the case of at least 10 families.^8^ Among these identified mothers or expecting mothers, 51 percent identified as being of Indonesian descent, 38 percent were of Filipino descent, and 11 percent reported having another ethnicity or combination of ethnicities. A notable feature of our sample is that the majority of mothers, despite being non-Malaysians, were either born in or had long settled in the country. Nearly 1 in 3 women were born within Malaysia, while only 8 women had lived in the country 10 years or less cumulatively. Mother respondents fell within the age range of 18–100, with a mean age of 33.6 years. The 92 mothers had an average of 4 children, ranging from 1 to 13.^9^ Among the total number of children reported in the study, 37 percent were born in a healthcare facility, while 63 percent were born at home (Table 2).

**Table 2:** Descriptive statistics of mothers (*N* = 92)

Age	*N* of years living in Malaysia	Ethnicity	*N* of children
18–31: 26	1–10: 8	Filipino: 38	1–2: 40
31–44: 14	11–20: 24	Indonesian: 51	3–4: 25
44 and over: 8	21–30: 11	Other: 3	5 and up: 27
No response: 44	31 and up: 5		
	Born in Malaysia: 25		
	No response: 19		

### The integrated three delays model

**Fig. 1:**
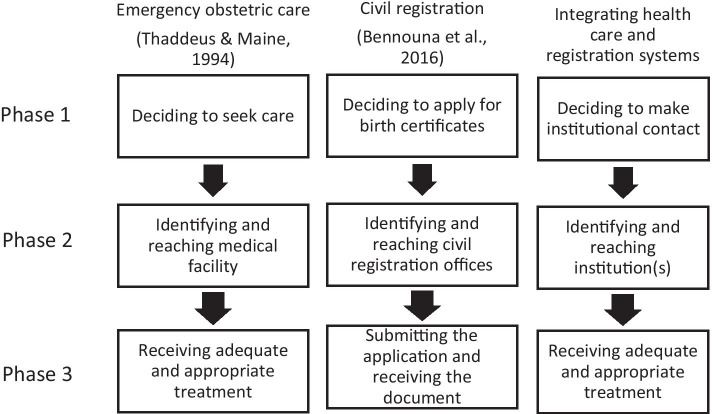
Comparing adaptations of the three delays framework

Based on the thematic coding of our qualitative data, we propose an integrated three delays framework that synthesizes and extends upon Thaddeus and Maine (1994) and also takes inspiration from Bennouna et al.’s (2016) adaptation of their original framework to the realm of civil registration (see Fig. 1). We first review the original three delays model to better exemplify how our framework builds upon their theoretical intervention. To make sense of the factors contributing to maternal mortality, Thaddeus and Maine (1994) organize the obstacles to obtaining quality maternal health care following the onset of an obstetric complication into three main stages. Phase I delays occur when deciding to seek care in the first place. Individuals and families take various factors into account when deciding whether to attempt to access a healthcare institution, which Thaddeus and Maine (1994) categorize as “sociocultural factors,” as well as perceptions about both the “accessibility of services” and “quality of care” provided by the medical facility. Sociocultural factors include, for example, educational attainment or constraints on women’s rights and autonomy. Perceptions about accessibility and quality of care are shaped by considerations such as cost of transportation and services, and distance to the facility. Phase II delays occur when potential patients attempt to reach the point of care. Even when a woman or family does decide to visit a health facility, factors, such as geographical distance, poor roads, or lack of transportation may lead to a failure in doing so. Phase III delays occur in the process of receiving adequate treatment once the individual or family reaches the healthcare facility. Shortages in trained personnel, inadequate facilities, or poor quality of care also contribute to maternal mortality risk (Fig. 1).

By transposing the three delays framework to the study of civil registration access in Indonesia, Bennouna et al. (2016) demonstrate its striking cross-sectoral utility. Based on a series of focus groups conducted across four Indonesian districts, they sought to identify barriers obstructing Indonesian parents from registering the births of their babies. Similar to the original framework, Phase I delays in the context of birth registration originate in deciding to apply for a birth certificate in the first place. The decision to apply for a birth certificate, such as the decision to use a healthcare facility, is influenced by a combination of socioeconomic and cultural factors as well as perceptions of both accessibility to government offices and the quality of services provided there. Here, the authors describe socioeconomic and cultural factors as including financial constraints, perceptions and awareness of the importance of birth registration, a consideration of the opportunity costs involved in making the effort to undergo the birth registration process, as well as religious and cultural customs surrounding naming practices, among other factors. Phase II delays that families might face include being able to identify and reach civil registration offices, which, as with reaching healthcare facilities, may be affected by factors such as geographical distance and availability of transportation. Finally, Phase III delays, analogized from those faced when trying to receive adequate and appropriate medical care at the facility, can occur in the process of submitting birth registration applications at the civil registration office. Factors affecting the successful lodging of applications include individuals’ abilities to navigate potentially complex administrative procedures to produce error-free submissions, as well the “friendliness, helpfulness, and competence of human resources” (Bennouna et al., 2016, p. 5).

Particularly instructive was Bennouna et al.’s (2016) observation about the striking applicability of best practices for reducing barriers to obstetrical care to the task of reducing barriers to civil registration. They, furthermore, single out the role of healthcare providers and systems for facilitating the uptake of birth registration, noting how Indonesia’s “civil registration system parallels and often intersects with the primary healthcare system, creating opportunities for the two systems to mutually reinforce one another” (p. 12). Indeed, giving birth in the presence of a skilled health attendant, as well as making use of postnatal care, was found to be positively correlated with children’s possession of birth certificates in the Indonesian context (Jackson et al., 2014).

**Fig. 2:**
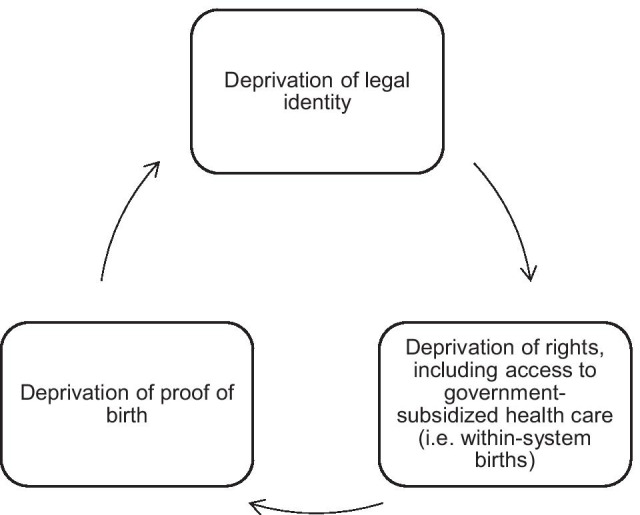
The intergenerational cycle of exclusion from civil registration and healthcare systems

In this study, we heed such recommendations about the importance of contact with healthcare institutions as a determinant of birth registration by explicitly modeling access to maternal health care and birth registration as cyclical co-determinants of one another, using the case of LMPs in Sabah, Malaysia. We venture that improving access to health care and improving governments’ ability to monitor population health via expanding civil registration coverage are mutually constitutive goals: Incomplete civil registration systems make it difficult to produce accurate vital statistics and population health indicators, such as infant and maternal mortality. Without vital documents, such as birth certificates, individuals, may face challenges establishing a legal identity, without which it may become difficult to regularize one’s status or even claim a nationality. The deprivation of legal identity is associated with an inability to enjoy its associated rights and benefits, including health care. Exclusion from healthcare systems raises the risk of mortality and morbidity, as well as the probability that such events play out beyond the knowledge of governing bodies. Out-of-system pregnancies and births increase infants’ risk of going unregistered, which can perpetuate an intergenerational cycle of exclusion (Fig. 2).

Our extension of the three delays framework brings to light previously undertheorized causes of delays to accessing both maternal health care and birth registration: those stemming from legal and political factors. Focusing on the experiences of LMPs in the Malaysian context compels us to avoid taking for granted the notion that access to basic rights, such as healthcare and identity documentation, are allocated on the basis of legal status. While Thaddeus and Maine (1994) explicitly sought to move away from individual-level explanations that blame people for their “ignorance, illiteracy, poverty, laziness, or superstition” to instead recognize the informed calculations individuals make in response to systemic shortcomings, their framework does not take into account the role of the state in creating unequal access to health care across different populations (p. 1100).

More recent research about maternal healthcare access, much of which is guided by the three delays approach, also privileges social, cultural, and economic barriers (Gabrysch & Campbell, 2009). This may be because the populations in question in these studies are composed of citizens only, which renders legal barriers less salient. However, even studies exploring foreign-born populations’ reproductive healthcare usage similarly emphasize sociocultural factors, such as language barriers, different norms and values between host and sending contexts, or deficiencies in information and knowledge⁠ (Binder et al., 2012; De Freitas et al., 2020; Esscher et al., 2014). It is important to recognize that typically, not all residents of a country enjoy equal legal entitlement to government health services. Furthermore, with an estimated 79.5 million people having fled their homes by the end of 2019, migrant-receiving states today are contending with populations that are territorially present within countries despite being excluded from national membership (UNHCR, 2020). By focusing on LMPs, a population that experiences significant degrees of legal marginalization, we demonstrate how state policies both directly and indirectly stratify access to rights and benefits—namely, a legal identity and health care.

**Fig. 3:**
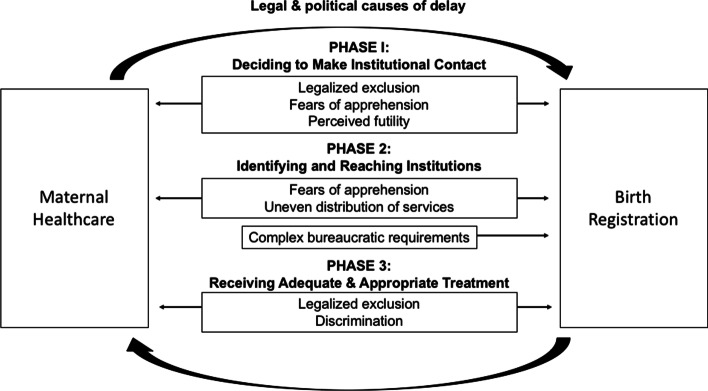
The integrated three delays model

Our three phases of delay closely mirror those identified by Thaddeus and Maine (1994) and Bennouna et al. (2016) with regard to maternal health care and birth registration, respectively: Phase I delays occur when LMPs decide to make contact with a government institution (health or registration) in the first place. Phase II delays are those that occur when identifying and reaching the targeted institution. Phase III delays are those that impede the reception of adequate and appropriate treatment at the healthcare facility or registration office. Where our framework departs, however, is in its identification of legal and political, rather than socioeconomic or cultural, factors determining institutional contact and the ensuing outcomes from these interactions, which we describe in detail in our report of our findings below (Fig. 3).

### Phase I: deciding to make institutional contact

Deciding to seek care is the first potential site of delay in making contact with healthcare institutions following the onset of an obstetric complication (Thaddeus & Maine, 1994) or in deciding to access prenatal care from or give birth in the presence of skilled attendants (Gabrysch & Campbell, 2009). Correspondingly, the first potential site of delay in registering a birth lies in whether the child’s parent decides to initiate the process, according to Bennouna et al. (2016). In the integrated three delays model, decisions to seek out maternal health care and birth registration by LMPs in Sabah are conditioned by similar delay factors. We show how exclusion from one system discourages families from attempting to make contact with another one. We focus on the political and legal factors that disincentivize institutional contact and, in doing so, we draw attention to the structural conditions that constrain individuals’ decision-making processes. Study participants commonly highlighted two major considerations that they took into account when deciding to make contact with either health or registration systems in Sabah: their legal ineligibility to receive publicly subsidized health care or birth certificates, and a fear of apprehension or being turned away. For many, both considerations amounted to perceiving a sense of futility in being able to receive adequate treatment from Malaysian governmental authorities.

#### Legalized exclusion

Exclusion from civil registration systems jeopardizes individuals’ ability to secure a legal identity: A birth certificate is often the foundation upon which individuals secure other important forms of identification, such as national identity cards, driver’s licenses, and passports. Providing official proof of individuals’ parentage and place of birth, birth certificates also function as evidentiary bases for determining citizenship and, therefore, eligibility for its associated rights, such as health care.

The primary reason that households cited for having out-of-system pregnancies and births was the high cost of visiting government clinics and hospitals for non-citizens owing to a lack of citizenship status. While the Malaysian government has been committed since independence to expanding its healthcare infrastructure nationwide, access continues to be heavily stratified according to legal status. Today, Malaysian nationals enjoy a universal public healthcare system, which is complemented and rivaled by a competitive private sector (Loganathan et al., 2020). Outpatient treatment at a public clinic or hospital costs a nominal fee for citizens, with surgeries and other procedures heavily subsidized (Kementerian Kesihatan Malaysia, 2020). It is estimated that 98.9 percent of births in Malaysia occur in a healthcare facility, according to the latest figures from 2014 (UNICEF, 2020b).

In contrast, non-citizens are largely priced out of healthcare access in Malaysia due to their exclusion from the public safety net. This includes the country’s sizable population of overseas foreign workers, who, even if in possession of valid visas, are not eligible for government-subsidized health care.^10^ Stateless persons born in the country are functionally regarded as foreigners and, therefore, also denied eligibility for subsidized care in most situations. This population includes not only individuals who are *de jure* stateless, but also those who may have a legitimate claim to Malaysian nationality but are unable to substantiate these claims through the administrative or judicial system.

Financial barriers to accessing health systems arising from a lack of citizenship status have increased dramatically in recent years. In January 2016, non-citizens’ access to hospital fee subsidies were eliminated 2 years earlier than initially pledged (Loganathan et al., 2020). At the end of 2014, then-Prime Minister Najib promised that fees would rise only incrementally to this “full” rate from 2015 to 2018. On the 2016 hike in fees for foreigners, an obstetrician noted during an interview, “In the last 2 years they’ve changed the policy so now non-citizens have to pay more. A lot of people without documents don’t come for antenatal care, or they go to a cheap general practitioner who may not be experienced in antenatal care. They might opt out of doing ultrasound scans because they have to pay extra for that.”

Indeed, many interview participants expressed strong preferences for giving birth outside of a formal medical facility, with a common alternative being giving birth with the attendance of a *bidan kampung* (a traditional midwife). Reliance on traditional midwives during both pregnancy and birth was a common strategy for women seeking to avoid costly institutionalized care completely. While *bidan kampung*, who often were elderly women from the coethnic community, played important and respected roles, they tended to lack professional training and certification, and often  practiced beyond the regulatory reach of the Ministry of Health. Exclusion from formal healthcare providers has not only exposed women who are LMPs to health risks, but has also meant that their pregnancies and births take place without the proper documentary proof that these had occurred in Malaysia.

According to existing literature, the decision to have home births might typically be attributed to sociocultural factors, such as distrust of mainstream institutions by rural-based or uneducated mothers. However, it was not uncommon for women to have given birth inside a medical institution in their home countries, or prior to the hiking of foreigner fees in Malaysia, and then later having to resort to home births due to financial constraints. These patterns suggest that migrant women might have opted for within-institution births if it had been financially feasible to do so. For example, one Timorese Indonesian mother of four gave birth to her first two children in a government hospital in Sabah at a time when hospital fees were still manageable. Her third and fourth children, however, were born at home after it became unaffordable to give birth at the hospital.

Other women referenced instances of relatives or neighbors struggling with financial burdens after giving birth in a hospital, which informed their own decisions to attempt home births. One Filipino Muslim woman recounted how her daughter-in-law was invoiced 3000 Ringgit ($750 USD) by the government hospital for her emergency breech delivery. She stated that even vaccinations, which cost 40 Ringgit ($10 USD) each for non-Malaysians, would be difficult to afford for poor migrants, such as herself.

One pregnant Timorese Indonesian, who was 19 years at the time of our interview, was determined to give birth with the assistance of a *bidan* and would only go to the hospital in the case of an emergency during labor. She alluded to the compounded difficulties of being not only a foreigner but one without legal status: “Because we do not have passports, we need to pay an expensive fee. It’s so hard without a passport.” Witnessing or hearing rumors about family, friends, and neighbors being served exorbitant bills by hospitals—and, in some cases, nevertheless having adverse pregnancy outcomes—served to discourage some interview participants from frequenting clinics and hospitals themselves.

Financial barriers arising from their legalized exclusion exerted a comparatively more indirect influence on households when it came to birth registration. LMPs who are mothers are disincentivized from registering their children’s births in part by the high opportunity costs associated with attempting to do so. To reiterate an earlier point, in the Malaysian context, birth registration is not an automatic process. That is, the onus falls upon the child’s parents to travel to the National Registration Department to apply for a birth certificate. Extensive and stringent documentary evidence is required to register a birth; without this evidence, applications are denied (see Table 1).

Organizations, such as UNICEF, Plan International, and the World Bank, have advocated for the elimination of late fees for birth registration, arguing that while these fees are intended to incentivize the timely registration of births, they often result in penalizing and further marginalizing impoverished families (Plan International, 2014; UNICEF, 2013; World Bank Group, 2018). Indeed, such amounts might prove to be prohibitive for LMPs in Sabah, who live, as some interviewees described it, “from hand to mouth.” More commonly, however, LMPs we talked to were constrained in their ability to register their children’s births by the anticipated opportunity costs involved. For households living outside of Sabah’s cities and towns, where National Registration Department offices are concentrated, birth registration is a costly endeavor. Laborers employed in the palm oil industry—in which wages are earned according to the amount of crop harvested—find it particularly difficult to afford the time away from work as well as transportation costs to the nearest civil registration office.

#### Fears of apprehension

The second major deterrent in deciding to make contact with health or registering institutions was a well-founded fear of arrest, detention, and deportation due to a lack of legal status. Stateless persons, despite having been born in Malaysia, experience a lived reality similar to that of “illegal” immigrants in that their lack of nationality and/or documentation to prove it renders them targets of suspicion in the eyes of authorities. Interview participants were fearful of the risks involved in approaching hospitals or National Registration Department offices, which are typically located in towns and cities. Visiting a hospital or a registration office would require them to venture beyond their spaces of residence and employment and into the public eye. Registration departments in Sabah were particularly regarded as risky places, because some are housed in Urban Transformation Centres, which are one-stop complexes that house a number of government services, including the Immigration Department and the police.

For example, Maria, a stateless woman of East Timorese descent who was pregnant for the first time, was discouraged by her mother-in-law from planning to give birth at the hospital in the nearest town. Maria was born in Sabah to undocumented immigrants from Indonesia who had come to Sabah to work on a palm oil plantation. Without a birth certificate or any other form of documentation, and having never been to Indonesia, Maria felt that her stateless status is not reflective of her affective ties to Malaysia. Maria decided to heed her mother-in-law, an Indonesian migrant worker with an expired visa, who feared that Maria would get *ditangkap* (Malay for “caught” or “arrested”) by the authorities on the way to or even at the hospital. The concern among irregularly statused households that hospitals could function as surveilling agents is a legitimate one. In September 2001, the Ministry of Health released a circular to state health directors outlining guidelines for the reporting of *pendatang tanpa izin* (a derogatory term in Bahasa Malaysia for unauthorized immigrants) to the police by all staff members employed by the Ministry.^11^⁠

#### A perceived sense of futility

Contrary to the commonly expressed belief among development agencies and even scholars that marginalized families do not apply for birth certificates for their children out of ignorance, the families we spoke to were highly aware of the importance of securing their children’s legal identity through birth registration. The lack of documentation and legal status constrained LMPs in many aspects of their lives, serving as constant reminders of the stakes involved in failing to obtain documentation for their newborn children. Likewise, pregnant mothers placed high value on formal prenatal and obstetric services, regardless of whether they were able to afford them. Rather than explain away unregistered births and out-of-system pregnancies as consequences of lack of knowledge or awareness on behalf of uneducated and impoverished parents, we instead draw attention to the political and legal forces that constrain individuals’ abilities to act in their own and on behalf of their families’ best interests.

Legalized exclusion from the public safety net and fears of arrest informed LMPs’ perceptions of their ineligibility for accessing both health care and registering services due to their irregular status. “It’s hard for non-citizens,” said Mariana—a young expectant mother who was born in Indonesia but brought to Sabah as a child—with regard to accessing hospitals. “Even if you have a valid passport, you need to pay an expensive fee to go to the hospital,” she reflected, going on to note that she did not even have a passport, valid or expired. Another Indonesian migrant couple explained that they did not have birth certificates for their youngest three children, because they were all born at home. Without proof from the hospital that their children were in fact born on Malaysian soil, the couple knew that any attempts to apply for birth certificates for them would be refused by the National Registration Department.

Knowledge about legal eligibility for subsidized health care, and about the documentary requirements for registering births in Malaysia, were circulated via social and familial networks. Families also acquired awareness of their legal and bureaucratic constraints through failed firsthand attempts at applying for birth certificates or accessing health services. In the next section, we discuss the major challenges that LMPs encountered upon deciding to make institutional contact with public healthcare providers or registering bodies.

### Phase II: identifying and reaching institutions

Thaddeus and Maine (1994) define Phase II delays as barriers to identifying and reaching healthcare facilities after an individual or family has made the decision to visit one. Bennouna et al. (2016) observe that such delays are also relevant to the matter of birth registration and identify “information, infrastructure, and resources” as factors limiting Indonesians’ journeys to registration offices. We identify three major legal and political barriers that LMPs faced while attempting to register their children’s births (fears of apprehension, uneven geographical distribution of services, and complex bureaucratic requirements), which we elaborate upon below.

#### Fears of apprehension

In the foregoing section discussing Phase I delays, LMPs were discouraged from venturing into public spaces due to fears of arrest, detention, and possible deportation. For those who decide to take the risk—for example, in cases of health emergencies—potential apprehension presents itself at every step of the journey, rendering illegality a major constraint to geographic mobility.

Roadblocks are one strategy employed by the Malaysian authorities for finding unauthorized immigrants. In 2020, after the outbreak of the COVID-19 pandemic, the government launched Ops Benteng, a joint operation involving the Armed Forces, the Royal Malaysian Police, the Malaysian Maritime Enforcement Agency, and the Malaysian Border Control Agency to “ensure the country’s borders are safeguarded, and to eliminate cross-border crimes and to prevent the spread of COVID-19.”^12^ Since its launch in May 2020, hundreds of roadblocks have been staged nationwide, with over 7,000 immigrants having been rounded up for deportation as of October 2020 estimates.^13^

#### Uneven geographical distribution of services

The spatial distribution of public services in relation to the residential patterns of LMPs is not random or accidental but politically designed. In this section, we note that the Malaysian National Registration Department is not the only governmental body that has registering capacities. People of Filipino and Indonesian descent can theoretically avail themselves of civil registration and passport services provided by their countries’ consulates. We describe this access as theoretical, because it is not feasible for the vast majority of immigrants and their families to bridge the geographical gap that separates foreign nationals from their consular representatives.

In the case of Indonesian migrants, the Indonesian government has two consular offices in the state of Sabah: one in the capital city of Kota Kinabalu, and the other on the opposite coast in the city of Tawau. Indonesian migrant workers, particularly those living on remote palm oil plantations in the interior of the state, are often unable to travel to these urban centers for the various legal and resource-related reasons already discussed. To expand their service coverage, Indonesian consular representatives often arrange outreach missions to remote plantation areas to conduct campaigns to supply Sabah-born children of Indonesian descent with documents called *akta kelahiran* (birth certificates). These efforts are supported by local civil society actors, who conduct much of the local grassroots organizing prior to the arrival of consular missions. However, the demand for consular assistance far outpaces the frequency of these missions, leaving families without consular assistance for months at a time or potentially without ever receiving assistance.

The Filipino consulate also conducts regular outreach missions on a rotating schedule of locations across Sabah, which are commonly learned about by word of mouth or on social media. In contrast with the Indonesian government, however, there is no permanent diplomatic presence of the Philippines on Sabahan soil owing to long-standing competing political claims over the territory of Sabah between the Filipino and Malaysian governments (Fernandez, 2007; Samad & Bakar, 1992).⁠ Interview participants reported waiting months before they were able to receive consular assistance, including birth registration. Even when an outreach mission did take place within their locality, and they were fortunate enough to have been notified of its occurrence, families often contended with long lines due to the high demand for assistance among fellow overseas nationals.

#### Complex bureaucratic pathways

Previous applications of the three delays frameworks have conceptualized Phase II delays as occurring across spatial dimensions. Here, “journeys” to sites of public service delivery (more so for civil registration offices than for healthcare institutions, in the Malaysian case) were bureaucratic in addition to geographical. For legally marginalized populations seeking to obtain birth certificates for their children, the process is often circuitous and riddled with dead ends, resulting in long delays or unsuccessful registration outcomes.

To obtain a Malaysian birth certificate, families must be able to furnish several pieces of supporting documentary evidence (see Table 1). Even one missing document can set off an intergenerational chain reaction of undocumented status. Parents of children who were gestated and born outside of a healthcare facility were often turned away from National Registration Department offices, because they lacked prenatal cards, infant health records, or immunization records. Without these, civil registration officers could argue that applicants lack sufficient evidence of the child having been born on Malaysian soil.

The possession of marriage certificates by parents posed another bureaucratic barrier to birth registration. One Indonesian migrant recounted that she could not register her four children’s births, because even though three of them had been born in a government hospital, she and her husband did not formally register their marriage with any government authority. She further elaborated that they were unable to obtain a marriage certificate, because her husband’s work visa had already lapsed at the time.

In summary, families were required to embark on labor- and resource-intensive journeys to obtain a birth certificate. Given that this one document is conditional upon the possession of a range of documents, which are themselves conditional upon the possession of other documents, there were myriad potential causes of delay or failure to register a birth. Civil registration systems are, therefore, interlocked with and dependent on a range of other institutions, including religious bodies, consular officials, and particularly healthcare facilities.

### Phase III: receiving adequate and appropriate treatment

#### Legalized exclusion and individual discrimination

This study has alluded to the fact that reaching a government service provider does not guarantee adequate receipt of services. Thaddeus and Maine (1994) highlighted factors such as insufficient healthcare infrastructure and personnel, lack of training among care providers, and sociocultural differences as reasons for poor treatment of patients experiencing obstetric complications once they have reached a healthcare facility. In identifying Phase III barriers to civil registration, Bennouna et al. (2016) drew attention to factors such as the severe consequences of clerical errors made on behalf of applicants or officers, as well as resource limitations of offices that were routinely understaffed and undersupplied.

In Sabah, we found that LMPs are denied adequate and appropriate treatment at healthcare and registering facilities due to both individual and institutionalized discrimination. We discuss forms of individual discrimination as they play out through the discretionary powers held by frontline service providers (Lipsky, 1980). By institutionalized discrimination, we refer to the kinds of prejudicial treatment embedded in laws, policies, and administrative practices. While such institutional processes may be ostensibly color blind, they are discriminatory in their impacts by engendering unequal access to opportunities and resources (Pager & Shepherd, 2008).

Without valid legal status, LMPs in Sabah were at times met with threats of arrest upon arrival at healthcare facilities or registering offices, though such occurrences were comparatively rare in our fieldwork. Hasnia, a Sabah-born stateless woman of Filipino descent, gave birth to a boy in 2011. The boy’s father, a member of the *Orang Sungai*, an Indigenous group in Sabah, went to the National Registration Department and managed to successfully register the boy’s birth. However, their lack of a marriage certificate meant that the boy would follow the nationality of his mother—*bukan warganegara* (non-citizen), as she is a non-citizen according to her birth certificate. When it came time to enroll her son in primary school, his birth certificate was allegedly confiscated by an education officer who questioned the boy’s right to attend a government school. By then, the boy’s father had become estranged from the family, leaving Hasnia the sole caregiver to their son. When Hasnia went to the National Registration Department and attempted to obtain an extract of her son’s birth certificate, bringing along with her a photocopy of the original, she was, according to her recollection, accused by the officer of attempting to fraudulently obtain a birth certificate. Without legal status of her own and thus fearing for her safety, Hasnia fled the office and has avoided government officers ever since. Her son remains without possession of his birth certificate.

While frontline healthcare providers, such as doctors and nurses, tended to be less discerning of patients’ legal status due to a professional obligation to provide care to the best of their ability, hospital administration staff faced greater pressure to assess individuals’ ability to afford care. People of Filipino and Indonesian descent, especially those with lower socioeconomic status, contend with presumptions of undocumented status in their everyday interactions, a process that scholars have described as the “racialization of illegality” [for a recent review, see Menjívar (2021)]. That is, in the Sabah context, Filipino or Indonesian racial identity has become inextricably associated with illegality, with race becoming a proxy for legal status and consequently eligibility for public services. For example, Yohana, an Indonesian-Timorese mother, recalled the occasion when she attempted to visit a government hospital for a routine prenatal checkup. Despite insisting that she was lawfully present in the country, she ended up being taken to the police station on the suspicion that she was an “illegal.” Yohana was released only when her Malaysian employer came to pick her up at the station with her papers.

Other participants reported being able to give birth at government hospitals but were saddled with hefty fees afterwards. Wati, an Indonesian woman with an expired passport, recalled being charged 1700 Ringgit ($425 USD) for a normal birth and a 1-day stay at the hospital, which she estimated to be equivalent to around 3-month salary for her household. Though her family is striving to pay off their debt to the hospital in instalments, Wati harbors shame from the experience. “People can still give birth at the hospital if their passport has expired, but the staff at the hospital will be angry,” Wati recalled when describing the belittling treatment she received as an undocumented patient. “They will ask, ‘Why did not you renew your passport?’”

Even when frontline workers are properly doing their jobs, formal laws and administrative procedures work to perpetuate the disadvantage and exclusion of LMPs. In our discussion of Phase I delays, we detail study participants’ sense of futility in being able to register their children or afford public health services due to insufficient documentation. This anticipation of rejection is not merely borne out of conjecture. As we show in our section on Phase II delays, households expended significant amounts of time, energy, and money trying—and often failing—to satisfy government agencies’ documentary requirements. Delays in Phase III stem from the stringent bureaucratic requirements that are enforced at the National Registration Department and other governmental bodies, which structurally disadvantage legally marginalized populations.

In addition to passports and visas, participants commonly identified prenatal health cards as an essential document, as well as one that was difficult to obtain. Being in possession of the full repertoire of documents would require women not only to give birth inside a healthcare facility, but also to be in regular contact with healthcare providers throughout the prenatal stage. In our ethnographic observations, we witnessed parents being summarily turned away from National Registration Department counters if they did not possess all the documents required for birth registration, even if they had brought their babies with them. The high burden of documentary proof demanded by the National Registration Department stems from political pressures to safeguard Malaysian citizenship from “illegal” immigrants. The politicization of birth registration in Sabah, however, has led to the deprivation of the fundamental right to a legal identity among children born on Malaysian soil.

## Discussion and conclusion

The “inter-linkages and integrated nature of the SDGs” reflect the belief that poverty eradication with the aim to “leave no one behind” requires a holistic and inclusive approach (United Nations, 2015). The achievement of any one target is incumbent upon the achievement of others, and the target to provide universal identity to all, including birth registration, is no exception. This study adapts Thaddeus and Maine’s (1994) three delays framework to model the interrelated barriers to maternal health care and birth registration among LMPs in Sabah, Malaysia—specifically, stateless persons, irregular migrants, and their descendants. Drawing on original ethnographic fieldwork conducted in Sabah, we argue that improving maternal health and expanding civil registration coverage are interdependent efforts. Exclusion from one system raises the risk of exclusion from the other, resulting in a range of negative consequences. Legal and financial barriers to accessing prenatal and obstetrical care increase the likelihood of out-of-system births by non-citizens, leaving their children at risk of being unaccounted for in Malaysia’s civil register. This not only compromises the government’s ability to monitor and improve its population health, but also places already marginalized mothers and children at greater risk of suffering negative health outcomes. Without proof of the circumstances of their births, children born to LMPs are unable to obtain birth registration and, therefore, may be deprived of a legal identity and even a claim to a nationality. These deprivations perpetuate an intergenerational feedback loop, wherein restrictive healthcare policies produce undocumented populations, who may pass on their undocumented or stateless status—and their associated ineligibility for public health services—to their children.

This being said, such experiences of exclusion do not affect all non-citizens equally or in the same way across Malaysia. The legally marginalized population in Malaysia is not homogenous, and their experiences and characteristics vary significantly. LMPs in Sabah have experiences that are distinctive from LMPs in Peninsular Malaysia due to the historical politicization within the state of immigration- and particularly documentation-related issues, as well as the protracted nature of citizenship exclusion among various communities. LMPs within Sabah itself can be further disaggregated into groups with distinctive vulnerabilities. Indonesian migrants, for example, have comparatively better access to Indonesian consular services in Sabah compared to Filipino migrants, whose government does not have a permanent consular presence in the state for political reasons. At the same time, persons of Filipino descent whose ancestors, or who themselves, had fled Mindanao to Sabah during the 1960s and 1970s are eligible for Sabah government-issued IMM13 cards as de facto recognition of their status as asylum seekers, which provides a modicum of rights that immigrants from other countries and time periods do not have. IMM13 eligibility and recognition, however, is temporary and is liable to be paused or revoked at the will of the state government. It is also important to distinguish between irregular migrants and their children who may be eligible to claim a nationality—for example, migrant workers whose visas have lapsed but who may still possess familial, cultural, or even documentary ties to their sending countries—and those in Sabah who are without any viable pathways out of being stateless—such as descendants of IMM13 refugees or undocumented foundlings. Thus, access to health care, birth registration, and other rights and protections must be considered through an intersectional lens and with consideration for the particular nature of legal exclusion that different non-citizen minorities face in Sabah and in Malaysia as a whole.

This leads to questions about the extent to which, and how, the integrated three delays model can be generalized beyond the Sabah context. Sabah may upon first glance seem like an outlier in terms of the number, long-standing nature, and vulnerability of its non-citizen members. However, this case is an example of broader global trends in recent years of rising international displacement, particularly among countries in the Global South (International Organization for Migration, 2019), as well as receiving states’ increasing preferences for precarious and temporary forms of membership over making available pathways to permanent residence or citizenship (Donato & Massey, 2016; Fernandez-Kelly & Massey, 2007). The integrated three delays framework provides CRVS researchers with theoretical tools for identifying political and institutional determinants of under-registration within populations of heterogeneous legal and nativity statuses.

Enhancing protections for the health of non-citizens and lowering administrative barriers to civil registration will contribute to improving maternal and infant health, empowering governments’ abilities to monitor population health, and reducing the occurrence of statelessness. Measures that governments can take to improve the right to health and to a legal identity include adopting more flexible and inclusive bureaucratic requirements for registering births and other events—such as community-based approaches to verifying the occurrence and characteristics of a child’s birth—to lower barriers to the recognition of LMPs. Both sending and receiving states should coordinate with one another to improve protections for the right to health among migrants, including overseas migrant workers, and their children. This includes providing affordable and accessible health insurance and expanding both emergency and preventative maternal health services to migrant families.

Research about the causes and consequences of exclusion from CRVS systems is rapidly growing in the Sustainable Development Goals era, garnering wide interdisciplinary interest. While our qualitative approach enables us to detail and trace the complex factors and processes that lead to under-registration or out-of-system births, our small sample size prevents us from generating any reliable statistical estimates of the scale or prevalence of these phenomena. Second, our model is based on a single case study and cannot necessarily be generalized to other cases without first making adaptations to suit their particular legal and administrative contexts or demographic compositions. That being said, our qualitative study is intended to complement well-established quantitative demographic research in this subfield and to serve as a call for the continued methodological diversification of inquiries into CRVS-related issues.

## Data Availability

The data generated and analyzed during the current study are not publicly available to protect study participants’ identities.

## Appendix: Sample interview guide topics for household document inventory

Date:

Town/Estate:

ID:

A. Information about the household members
  1. Year of birth
  2. Place of birth
  3. Race/Ethnicity
  4. Religion
  5. Marital status
  6. Years of education
  7. Occupational history
  8. Migration history
B. Documents
  Potential document types: birth certificate; notice of birth; identity card; passport; work permit/visa; marriage certificate; death certificate; adoption certificate; household registration list; school certificate; other.
  9. Who issued it?
  10. How did you obtain it?
  11. Did you face difficulties obtaining it?
  12. What did it cost you, if anything?
  13. Why did you obtain it?
  14. What do you use it for?
  15. When and how often do you use it?
  16. Where do you keep it?
  17. What value does it have to you?
C. Healthcare access
  18. Reasons for seeking care
  19. Barriers to receiving care
  20. Cost of most recent visit
  21. Health status/conditions?
  22. Method of birth delivery? (unassisted at home, public hospital, public clinic, private hospital, private clinic, traditional birth attendant or midwife, other?)
  23. Reasons for choosing this method
  24. Cost of birth delivery
  25. Did the birth attendant share information/assistance about birth registration?
  26. Was a record of birth provided? (Specify issuing body)
  27. Birth certificate obtained?
D. Other observations or notes
  28. Context and environment
  29. Family history/structure
  30. Migration history/plans
  31. Legal status/documentation
  32. Education
  33. Healthcare access and usage
  34. Social ties
  35. Work

## Footnotes

1 See Report of the Commission of Enquiry on Immigrants in Sabah (2014), accessible at http://legacy.sapp.org.my/rci/RCI-Eng.pdf.

2 Malaysia’s *Births and Deaths Registration Act 1957*, Part II, Section 7(1).

3 The National Registration Department also distinguishes between “delayed” birth registration (which occurs between the 15th and 42nd day after the birth of a child), and “late” registration (which is a registration attempt that is made beyond 42 days after the birth of a child). The fees directly associated with delayed and late birth registration are 5 and 10 Ringgit ($1.20 and $2.40 USD), respectively (Jabatan Pendaftaran Negara, 2020).

4 https://www.jpn.gov.my/images/pdf/perkhidmatan/BorangN1.pdf.

5 https://www.jpn.gov.my/images/pdf/perkhidmatan/JPNWN01A.pdf.

6 Given the potential sensitivity of the nature of documentation and legal status, we recommend that interviewers avoid asking directly about these topics, but more gradually transition into covering them after an extensive discussion about demographic information and life histories. Given the salience of documents and legal status, or rather the lack thereof, in Sabah-based LMPs’ everyday lives, these topics would routinely come up organically in conversation.

7 The household and family formations of LMPs were diverse and at times complex, with many households containing multiple generations, extended family members, and non-biologically related members. These summary statistics represent the characteristics of mothers, and their associated children, from whom the household interview captured the most information.

8 Reasons that warranted follow-up interviews or ethnographic observations included reinterviewing women after they had given birth, providing information about civil registration and other documentation-related issues that families were struggling with, and observing or assisting families as they applied for documentation, among others. Opportunities to have additional encounters with households depended on a number of factors, including whether we were able to build rapport and trust with research participants.

9 The mean is rounded to the nearest 1 and includes five children who were born during the study.

10 While documented foreign workers have been required to be covered under the Foreign Worker Hospitalization and Insurance Scheme since 2011, many barriers prevent foreign workers from experiencing sufficient health protections and equitable access to care (Loganathan et al., 2020).

11 Director General of Health Circular No. 10/2001: Garispanduan Melaporkan Pendatang Tanpa Izin Yang Mendapatkan Perkhidmatan Kesihatan di Hospital dan Klinik Kesihatan.

12 Senior Defence Minister Datuk Seri Ismail Sabri Yaakob, quoted during a press conference on 8 July 2020. Nuradzimmah Daim (2020), “1391 illegal immigrants detained in Ops Benteng since May 1,” *New Straits Times,* 8 July, https://www.nst.com.my/news/nation/2020/07/606923/1391-illegal-immigrants-detained-ops-benteng-may-1.

13 Senior Minister Datuk Seri Ismail Sabri Yaakob, quoted in Mazwin Nik Anis (2020), “Over 7000 illegal immigrants nabbed since Ops Benteng started in May, says Ismail Sabi,” *The Star,*16 October, https://www.thestar.com.my/news/nation/2020/10/16/over-7000-illegal-immigrants-nabbed-since-ops-benteng-started-in-may-says-ismail-sabri.

## References

[CR1] AbouZahr C, de Savigny D, Mikkelsen L, Setel PW, Lozano R, Lopez AD (2015). Towards universal civil registration and vital statistics systems: the time is now. The Lancet.

[CR2] Acciaioli G, Brunt H, Clifton J (2017). Foreigners everywhere, nationals nowhere: exclusion, irregularity, and invisibility of Stateless Bajau Laut in Eastern Sabah, Malaysia. Journal of Immigrant & Refugee Studies.

[CR3] Allerton C (2020). Invisible children? Non-recognition, humanitarian blindness and other forms of ignorance in Sabah Malaysia. Critique of Anthropology.

[CR4] Almeida LM, Cladas J, Ayres-de-Campos D, Salcedo-Barrientos D, Dias S (2013). Maternal Healthcare in migrants: a systematic review. Maternal and Child Health Journal.

[CR5] Asad AL, Clair M (2018). Racialized legal status as a social determinant of health. Social Science & Medicine.

[CR6] Baltazar MAK, Cheong AR (2021). Reaching stateless and undocumented communities during the COVID-19 pandemic: lessons from the grassroots humanitarian effort in Sabah, Malaysia. The Statelessness and Citizenship Review.

[CR7] Bennouna C, Feldman B, Usman R, Adiputra R, Kusumaningrum S, Stark L (2016). Using the three delays model to examine civil registration barriers in Indonesia. PLoS ONE.

[CR8] Binder P, Borné Y, Johnsdotter S, Essén B (2012). Shared language is essential: communication in a multiethnic obstetric care setting. Journal of Health Communication.

[CR9] Cavazos-Rehg PA, Zayas LH, Spitznagel EL (2007). Legal status, emotional well-being and subjective health status of Latino immigrants. Journal of the National Medical Association.

[CR10] Cheong, A.R. (2019). *Omitted lives: access to civil registration and its implications for inequality (Dissertation).* Princeton University

[CR12] Cheong AR, Massey DS (2019). Undocumented and unwell: legal status and health among Mexican migrants. International Migration Review.

[CR13] Cuadra CB (2012). Right of access to health care for undocumented migrants in EU: a comparative study of national policies. European Journal of Public Health.

[CR14] Dahan, M., & Gelb, A. (2015). The Role of Identification in the Post-2015 Development Agenda. *Center for Global Development*. https://www.cgdev.org/sites/default/files/CGD-Essay-Dahan-Gelb-Role-Identification-Post-2015.pdf

[CR15] De Freitas C, Massag J, Amorim M, Fraga S (2020). Involvement in maternal care by migrants and ethnic minorities: a narrative review. Public Health Reviews.

[CR16] de Smalen AW, Chan ZX, Abreu Lopes C, Vanore M, Loganathan T, Pocock NS (2021). Developing an evidence assessment framework and appraising the academic literature on migrant health in Malaysia: a scoping review. British Medical Journal Open.

[CR17] Dollah R, Abdullah K (2018). The securitization of migrant workers in Sabah, Malaysia. Journal of International Migration and Integration.

[CR18] Donato KM, Massey DS (2016). Twenty-first-century globalization and illegal migration. The ANNALS of the Academy of Political and Social Sciences.

[CR19] Esscher A, Binder-Finnema P, Bødker B, Högberg U, Mulic-Lutvica A, Essén B (2014). Suboptimal care and maternal mortality among foreign-born women in Sweden: maternal death audit with application of the ‘migration three delays’ model. BMC Pregnancy and Childbirth.

[CR20] Fagernäs S, Odame J (2013). Birth registration and access to health care: an assessment of Ghana’s campaign success. Bulletin of the World Health Organization.

[CR21] Fernandez ES (2007). Philippine-Malaysia dispute over Sabah: a bibliographic survey. Asia-Pacific Social Science Review.

[CR22] Fernandez-Kelly P, Massey DS (2007). Borders for Whom? The Role of NAFTA in Mexico-U.S. Migration. The ANNALS of the Academy of Political and Social Sciences.

[CR23] Gabrysch S, Campbell OM (2009). Still too far to walk: literature review of the determinants of delivery service use. BMC Pregnancy and Childbirth.

[CR24] Gelatt J (2016). Immigration status and the healthcare access and health of children of immigrants*: immigration status and children’s healthcare access. Social Science Quarterly.

[CR25] Grit K, den Otter JJ, Spreij A (2012). Access to health care for undocumented migrants: a comparative policy analysis of England and the Netherlands. Journal of Health Politics, Policy and Law.

[CR26] Hacker K, Anies M, Folb BL, Zallman L (2015). Barriers to health care for undocumented immigrants: a literature review. Risk Management and Healthcare Policy.

[CR27] International Organization for Migration. (2019). *Key Migration Terms*. https://www.iom.int/key-migration-terms

[CR28] Jabatan Pendaftaran Negara. (2020). *Normal Registration of Birth (Sabah)*. https://www.jpn.gov.my/en/core-business/birth/sabah/pen-kelahiran-biasa-sb-eng

[CR29] Jackson D, Wenz K, Muniz M, Abouzahr C, Schmider A, Braschi MW, Kassam N, Diaz T, Mwamba R, Setel P, Mills S (2018). Civil registration and vital statistics in health systems. Bulletin of the World Health Organization.

[CR30] Jackson M, Duff P, Kusumanigrum S, Stark L (2014). Thriving beyond survival: Understanding utilization of perinatal health services as predictors of birth registration: a cross-sectional study. BMC International Health and Human Rights.

[CR31] Kassim A (2009). Filipino refugees in Sabah: State responses, public stereotypes and the Dilemma over their future. Southeast Asian Studies.

[CR32] Kementerian Kesihatan Malaysia. (2020). *Hospital Charges*. http://www.hkl.gov.my/index.php/advanced-stuff/hospital-charges

[CR33] Koh SY (2017). Race, education, and citizenship: Mobile Malaysians, British colonial legacies, and a culture of migration.

[CR34] Korinek K, Smith KR (2011). Prenatal care among immigrant and racial-ethnic minority women in a new immigrant destination: exploring the impact of immigrant legal status. Social Science & Medicine.

[CR35] Lai SL, Tey NP (2021). Deficiency in civil registration and vital statistics reporting in remote areas: the case of Sabah, Malaysia. Journal of Population Sciences.

[CR36] Lasimbang HB, Tong WT, Low WY (2016). Migrant workers in Sabah, East Malaysia: the importance of legislation and policy to uphold equity on sexual and reproductive health and rights. Best Practice & Research Clinical Obstetrics & Gynaecology.

[CR37] Liew JCY (2019). Homegrown statelessness in Malaysia and the promise of the principle of genuine and effective link. Statelessness & Citizenship Review.

[CR38] Lipsky M (1980). Street-Level Bureaucracy. Dilemmas of the individual in public services.

[CR39] Loganathan T, Chan ZX, Pocock NS (2020). Healthcare financing and social protection policies for migrant workers in Malaysia. PLoS ONE.

[CR40] Lumayag LA (2016). A question of access: education needs of undocumented children in Malaysia. Asian Studies Review.

[CR41] Malaysia Department of Statistics. (2020). *Population and Demography*. https://www.dosm.gov.my/v1/index.php?r=column/ctwoByCat&parent_id=115&menu_id=L0pheU43NWJwRWVSZklWdzQ4TlhUUT09

[CR42] McAuliffe, M., Khadria, B., & Bauloz, C. (2019). *World migration report 2020*. IOM

[CR43] Menjívar C (2021). The racialization of “Illegality”. Daedalus.

[CR44] Mohamed Razali R, Nordin R, Duraisingam TJ (2015). Migration and statelessness: turning the spotlight on Malaysia. Pertanika Journal of Social Sciences and Humanities.

[CR45] Muzzi, M. (2010). *UNICEF Good Practices in Integrating Birth Registration Systems into Health Systems (2000–2009). Case Studies: Bangladesh, Brazil, The Gambia, and Delhi, India*. UNICEF. https://www.unescap.org/sites/default/d8files/knowledge-products/UNICEF-birth-registration-in-health-systems.pdf

[CR46] Onagoruwa A, Wodon Q (2021). Correlates of birth registrations in East and Southern Africa and implications for civil registration and vital statistics systems. Public Health.

[CR47] Ortega AN, Fang H, Perez VH, Rizzo JA, Carter-Pokras O, Wallace SP, Gelberg L (2007). Health care access, use of services, and experiences among undocumented Mexicans and other Latinos. Archives of Internal Medicine.

[CR48] Pager D, Shepherd H (2008). The sociology of discrimination: racial discrimination in employment, housing, credit, and consumer markets. Annual Review of Sociology.

[CR49] Plan International. (2014). *What if...every child was in the picture?: Civil registration and vital statistics: the case for investment*. https://plan-international.org/publications/what-if-every-child-was-picture#download-options

[CR50] Pye O, Daud R, Harmono Y, Tatat (2012). Precarious lives: transnational biographies of migrant oil palm workers. Asia Pacific Viewpoint.

[CR51] Reed MM, Westfall JM, Bublitz C, Battaglia C, Fickenscher A (2005). Birth outcomes in Colorado’s undocumented immigrant population. BMC Public Health.

[CR52] Sadiq, K. (2010). *Paper citizens: how illegal immigrants acquire citizenship in developing countries*. Oxford University Press

[CR53] Samad PA, Bakar DA (1992). Malaysia-Philippines relations: the issue of Sabah. Asian Survey.

[CR54] Setel PW, Macfarlane SB, Szreter S, Mikkelsen L, Jha P, Stout S, AbouZahr C (2007). A scandal of invisibility: making everyone count by counting everyone. The Lancet.

[CR55] Siagian C, Wandasari W, Sahputra F, Kusumaningrum S (2019). Strategic yet delicate: the dilemma of involving health workers in facilitating birth registration in Indonesia. BMC Health Services Research.

[CR56] Siddiqi A, Zuberi D, Nguyen QC (2009). The role of health insurance in explaining immigrant versus non-immigrant disparities in access to health care: comparing the United States to Canada. Social Science & Medicine.

[CR57] Thaddeus S, Maine D (1994). Too far to walk: maternal mortality in context. Social Science & Medicine.

[CR58] UN Inter-agency Group for Child Mortality Estimation. (2019). *Levels & Trends in Child Mortality: Report 2019*. https://www.unicef.org/media/60561/file/UN-IGME-child-mortality-report-2019.pdf

[CR59] UNHCR. (2020). *UNHCR: Figures at a Glance*. https://www.unhcr.org/figures-at-a-glance.html

[CR60] UNICEF. (2013). *Every Child’s Birth Right: Inequities and Trends in Birth Registration*. https://www.unicef.org/publications/files/Birth_Registration_11_Dec_13.pdf

[CR61] UNICEF. (2020a). *Birth Registration*. UNICEF Data. https://data.unicef.org/topic/child-protection/birth-registration/

[CR62] UNICEF. (2020b). *UNICEF Data Warehouse*. https://data.unicef.org/resources/data_explorer/unicef_f/?ag=UNICEF&df=GLOBAL_DATAFLOW&ver=1.0&dq=MYS.MNCH_INSTDEL.&startPeriod=1970&endPeriod=2021

[CR63] United Nations. (2015). *Social development for sustainable development*. https://www.un.org/development/desa/dspd/2030agenda-sdgs.html

[CR64] Wahab A (2020). The outbreak of Covid-19 in Malaysia: pushing migrant workers at the margin. Social Sciences & Humanities Open.

[CR65] World Bank Group. (2018). *Incentives for improving birth registration coverage: a review of the literature*. http://pubdocs.worldbank.org/en/928651518545413868/Incentives-and-Birth-Registration030518.pdf

[CR66] World Health Organization. (2014). *Strengthening civil registration and vital statistics systems through innovative approaches in the health sector: guiding principles and good practices*. https://www.who.int/healthinfo/civil_registration/crvs_meeting_dec2013_report.pdf

